# Ultrastructural features of psychological stress resilience in the brain: a microglial perspective

**DOI:** 10.1098/rsob.240079

**Published:** 2024-11-20

**Authors:** Fernando González Ibáñez, Jared VanderZwaag, Jessica Deslauriers, Marie-Ève Tremblay

**Affiliations:** ^1^ Axe Neurosciences, Centre de recherche du CHU de Québec-Université Laval, Québec, Québec, Canada; ^2^ Division of Medical Sciences, University of Victoria, Victoria, British Columbia, Canada; ^3^ Neuroscience Graduate Program, University of Victoria, Victoria, British Columbia, Canada; ^4^ Faculté de pharmacie, Université Laval, Québec, Québec, Canada; ^5^ Department of Molecular Medicine, Université Laval, Québec, Québec, Canada; ^6^ Neurology and Neurosurgery Department, McGill University, Montréal, Québec, Canada; ^7^ Department of Biochemistry and Molecular Biology, University of British Columbia, Vancouver, British Columbia, Canada; ^8^ Centre for Advanced Materials and Related Technology, University of Victoria, Victoria, British Columbia, Canada; ^9^ Institute on Aging and Lifelong Health, University of Victoria, Victoria, British Columbia, Canada

**Keywords:** microglia, psychological stress, resilience, cellular stress, electron microscopy, ultrastructure

## Abstract

Psychological stress is the major risk factor for major depressive disorder. Sustained stress causes changes in behaviour, brain connectivity and in its cells and organelles. Resilience to stress is understood as the ability to recover from stress in a positive way or the resistance to the negative effects of psychological stress. Microglia, the resident immune cells of the brain, are known players of stress susceptibility, but less is known about their role in stress resilience and the cellular changes involved. Ultrastructural analysis has been a useful tool in the study of microglia and their function across contexts of health and disease. Despite increased access to electron microscopy, the interpretation of electron micrographs remains much less accessible. In this review, we will first present microglia and the concepts of psychological stress susceptibility and resilience. Afterwards, we will describe ultrastructural analysis, notably of microglia, as a readout to study the mechanisms underlying psychological stress resilience. Lastly, we will cover nutritional ketosis as a therapeutic intervention that was shown to be effective in promoting psychological stress resilience as well as modifying microglial function and ultrastructure.

## 1. Introduction

Psychological stress is a major risk factor for the development of major depressive disorder (MDD) which is a leading cause of disability in the world and the main risk factor for suicide. The World Health Organization has identified MDD as a major global health concern. In Canada, the prevalence of MDD was 4.7% in 2012, with a lifetime prevalence of 11.3% [1]. Direct and indirect costs have been projected to be 12 billion Canadian dollars per year [2]. Stress is an evolutionary adaptation required for survival, but prolonged or sustained (i.e. chronic) response to stressful stimuli can become maladaptive [3]. Chronic stress may result from stressful situations like war [4], personal loss [5], violence [6], chronic illness [7] or financial burden [8]. Not everyone who is exposed to stressful situations develops depressive symptoms, suggesting that inter-individual differences may regulate the risk for the development of MDD [9]. The mechanisms underlying stress susceptibility comprise genetics, diversity of coping mechanisms, cultural differences, environmental factors, as well as an interplay between all of them. Resilience has been defined as a resistance or ability to recover from stressful challenges in a positive way. The neurobiological mechanisms of stress resilience and the biological basis for its diversity has garnered great research interest to better understand the successful adaptations to stress and to minimize the deleterious consequences of psychological stress and its chronic long-term effects on health, notably of the brain [10].

Along the lifespan, in both animal models and human studies, chronic stress has been linked to an increased susceptibility to neurodevelopmental, neuropsychiatric [11–17] and neurodegenerative [18–22] disorders. Identifying neurological changes related to stress exposure has provided key insights for further studies focusing on alternative therapeutic approaches to rescue and revert these changes. As a consequence, there is increasing interest in exploring, using various animal models, the biological basis of stress resilience, covering key aspects like genetics, diversity of coping mechanisms and environmental factors contributing to driving stress resilience phenotypes [23]. These mechanisms notably involve several brain cell types, including microglia. This review will begin by summarizing microglial involvement in stress and resilience. Next, we will demonstrate how studying the cellular and subcellular properties of microglia can be a tool for understanding the cellular basis of resilience. Lastly, as a proof of concept, we will demonstrate how microglial ultrastructural analysis was instrumental in a recent investigation into ketosis in the context of stress resilience.

## 2. Microglia, stress, and resilience

Microglia are the resident immune cells of the brain, which are involved in several physiological processes during development and across the lifespan, such as vascular formation and remodelling [24], blood-brain barrier (BBB) integrity [25], glial modulation [26], synaptic plasticity [27], neuronal activity regulation [28], as well as the elimination of damaged tissue and debris [29]. These physiological roles are notably mediated through inflammatory mediators (both anti- and pro-inflammatory cytokines) and phagocytosis [30]. Microglial alterations have been observed in several contexts of neurodevelopmental, neuropsychiatric and neurodegenerative disorders [31,32]. In fact, in many cases microglial dysfunction can exacerbate disease progression, making these cells promising targets for prevention and treatment [33].

Microglia are associated with the physiological response to stress notably by their contribution to the hypothalamus-pituitary-adrenal (HPA) axis and mediation of inflammation. The HPA axis is the primary biological mechanism underlying the regulation of acute or chronic stress responses [34]. The key output of this neuroendocrine axis is glucocorticoids (GCs) which bind to two types of nucleoreceptors (i.e. mineralocorticoid receptors and glucocorticoid receptors (GRs)) dispersed throughout the peripheral and central nervous system (CNS). GCs are considered the primary steroid regulator of microglial properties [35] as they modulate microglial reactivity and the local inflammatory environment via several pro-inflammatory pathways [35–38]. Depletion of GR in microglia from adult female mice and antagonism of GR in adult male mice were shown to alter microglial morphology, modify microglial ability to respond to stress, as well as impair synaptic plasticity in the hippocampus, as well as mitigate microglia-mediated neuronal remodelling in the prefrontal cortex [39,40]. Disabling neuron–microglia communication or depleting microglia (via fractalkine receptor knockout or blocking colony-stimulating factor-1 signalling, respectively) also decreases the neurobiological and behavioural impacts of stress [41–48]. These studies suggest that microglia are crucial players involved in the stress-related changes observed across the brain.

Immune function plays an important role in the adaptation of the brain to stress. Yet, excessive and sustained inflammation may be maladaptive and has been recognized as a relevant mechanism in the aetiology of MDD [49]. A subset of patients diagnosed with MDD displayed increased levels of inflammatory markers (e.g. C-reactive protein, interleukin (IL)-6 and tumour necrosis factor-alpha) compared with healthy controls [50–52]. Preclinical studies also strengthened the relationship between inflammation and depressive-like behaviours with the observation of sickness behaviour following an immune challenge in rodents [49]. This sickness behaviour mimics several domains of MDD symptomology, including anhedonia, sleep alterations, fatigue, changes of appetite and cognitive impairment [49,53]. As microglia are the main orchestrators of brain inflammation, they can also react to psychological stress via an increased production of inflammatory mediators that moderate disease progression. Preclinical evidence supporting this premise demonstrated that anti-inflammatory therapies (e.g. using minocycline) affect microglial function and improve depressive-related phenotypes in rodents [54–56]. Other preclinical findings revealed that neuron–microglia communication through fractalkine signalling (between neuronal fractalkine and its microglial receptor CX3CR1) is required to develop depressive-like behaviours in mice [41,42]. In addition, changes in inflammatory cytokines as a response to antidepressants (e.g. fluoxetine) have been reported in preclinical and clinical studies [57,58]. Reduced levels of interferon-gamma and increased levels of IL-10 were both observed in blood samples of patients treated with fluoxetine [59]. These findings support the idea that inflammation and MDD symptomology share common pathways and can interact together [53].

Many studies have focused on the contribution of microglia to the pathogenesis of MDD symptoms and MDD-related brain inflammatory changes. Some researchers raised the idea that MDD could be considered a microglial disorder [60]. This strengthens the idea that microglia are an interesting target for novel therapies that address psychological stress and its consequences. Still, further studies exploring the roles of microglia in the neurobiology of stress resilience are required in the field and will be useful to uncover new pharmacological strategies or lifestyle habits that may exert neuroprotective effects against stress-related diseases, such as MDD. Among the tools to study microglia, imaging approaches are effective for studying the roles of microglia notably in the mechanisms behind stress resilience. These techniques allow one to visualize and analyse microglial density and distribution among various CNS regions, which is useful in describing microglial intervention at a population level [61]. In addition, microglial morphology which is directly linked to their surveillance activity and many functions (e.g. phagocytosis and interactions with synapses) is highly informative [62,63]. Changes in microglial properties can facilitate the identification of specific brain regions involved in physiological processes of clinical relevance. Indeed, microglia are equipped to respond to a vast diversity of signals in their CNS environment: their density, distribution and morphology, as well as expression of these markers, adapt in response to various environmental changes such as psychological stress [64].

Psychological stress can cause irreversible and reversible changes to the brain [10,17]. Lately, the interest in elucidating the mechanisms involved in the reversible changes of stress has increased, as they were proposed to be more directly involved in the resilience to acute and chronic stress [65]. When discussing microglial resilience, it could be understood as the processes that either promote microglial health, which could be the absence of cellular stress features, the absence of physiological responses like an increased production of pro-inflammatory mediators or the cellular presence of features or microglial states that have been specifically observed in the resilient brain (e.g. branched microglia with small soma engaged in surveillance). Reversible microglial changes, such as modulation of inflammatory cytokines and morphological alterations, have been observed in resilient populations [66–68]. This is particularly relevant to microglial biology, since microglia adapt to their environment and can change their function and adopt specific physiological states. Advanced imaging techniques, including electron microscopy (EM) have been key in the identification and characterization of microglial states and their features.

In addition, studying subcellular features and inter-cellular contacts is very relevant for microglial involvement in stress resilience research. EM enables the visualization of microglia, their organelles and interactions with other cells and the extracellular environment at the nanoscale, providing a level of detail that cannot be achieved by light microscopy. EM has provided key insights into the central roles of microglia in health and disease [69], notably by improving our understanding of particular microglial functions, like synaptic elimination mediated by phagocytosis [27], which contributed to creating the field of neuroimmunoplasticity. As EM detects features that cannot be explored by light or fluorescence microscopy, it provides unprecedented insights, leading to the discovery of a state of microglia named the dark microglia (DM), defined by their ultrastructural features (e.g. markers of cellular stress), with an increased abundance notably following chronic stress [31,70]. Microglia can respond differently to psychological stress which can result in changes to one or many of their organellar numbers and ultrastructural characteristics. This guide intends to increase the toolkit available for ultrastructural analysis with the goal of detecting ultrastructural changes in microglia that can point towards cellular processes that are affected by stress or involved in psychological stress resilience. The analysis of organellar changes has been useful in identifying changes in cellular function in different cell types and organs [71,72]. Investigation of microglia with these techniques is currently lacking, requiring additional studies and ensuring an area of opportunity for the field of psychological stress resilience.

The field of microglial research is currently at its crossroads, as further investigations need to integrate the most accepted views and novel recommendations pertaining to microglial states and nomenclature in novel experimental designs [63]. While microglia have been mostly targeted for the study of vulnerability to stress, advanced microscopy technologies, such as EM, also need to be used to study microglial features associated with the physiological processes of resilience. In the next part of this review, we will discuss how ultrastructural analyses of microglia can be used to expand our knowledge of microglial involvement in stress resilience. By presenting a thorough characterization of the most common ultrastructural features of microglia, their organelles, common alterations and potential biological interpretation, we intend to bring EM closer to research groups that want to work with this technique.

## 3. Ultrastructural analysis as a readout to study stress resilience

**Figure 1.**
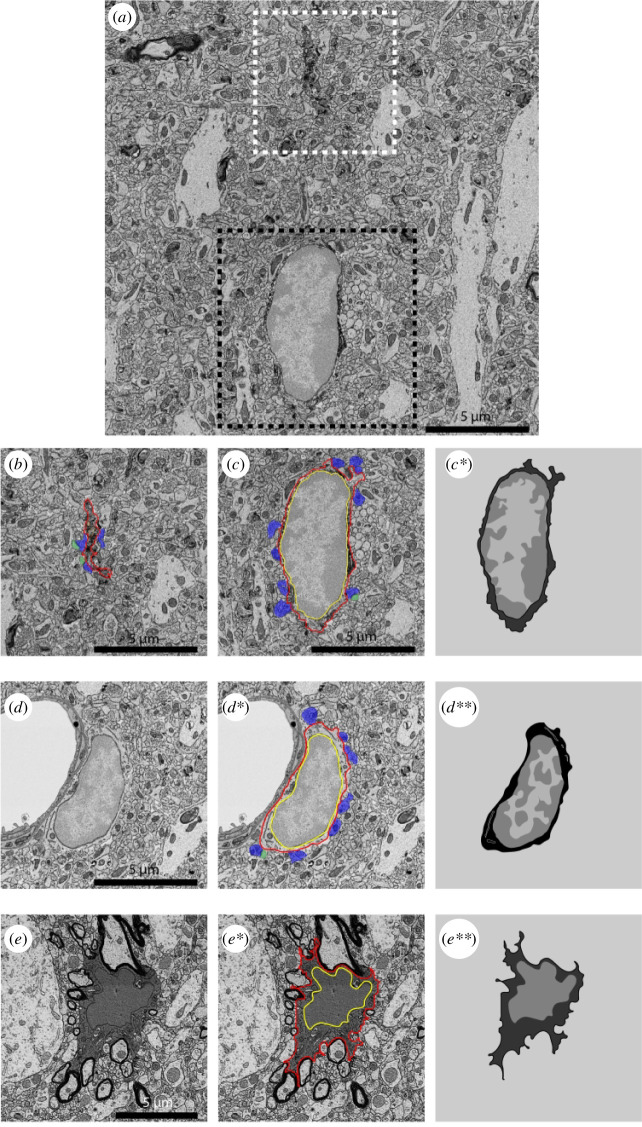
Representative 5 nm resolution scanning electron microscopy images depicting mouse microglia in the hippocampus. (*a*) An Iba1+typical microglial cell body (black dotted box) and an Iba1+microglial process (white dotted box). (*b*) Zoom in on Iba1+microglial process showing its inclusions and direct contacts to pre-synaptic axon terminals, post-synaptic dendritic spines and simultaneous contact to two synapse-forming elements classified as synaptic clefts. (c–d**) Typical microglia with an electron-lucent cytoplasm, long stretches of endoplasmic reticulum and a clear heterochromatin pattern. (e–e**) A dark microglia with an electron-dense cyto- and nucleoplasm, as well as diffuse heterochromatin pattern. Red outline: microglial plasma membrane; yellow outline: nuclear membrane; white outline: endoplasmic reticulum; orange outline: inclusions in microglial process; purple pseudocolouring: pre-synaptic axon terminal; green pseudocolouring: post-synaptic dendritic spine.

**Figure 2.**
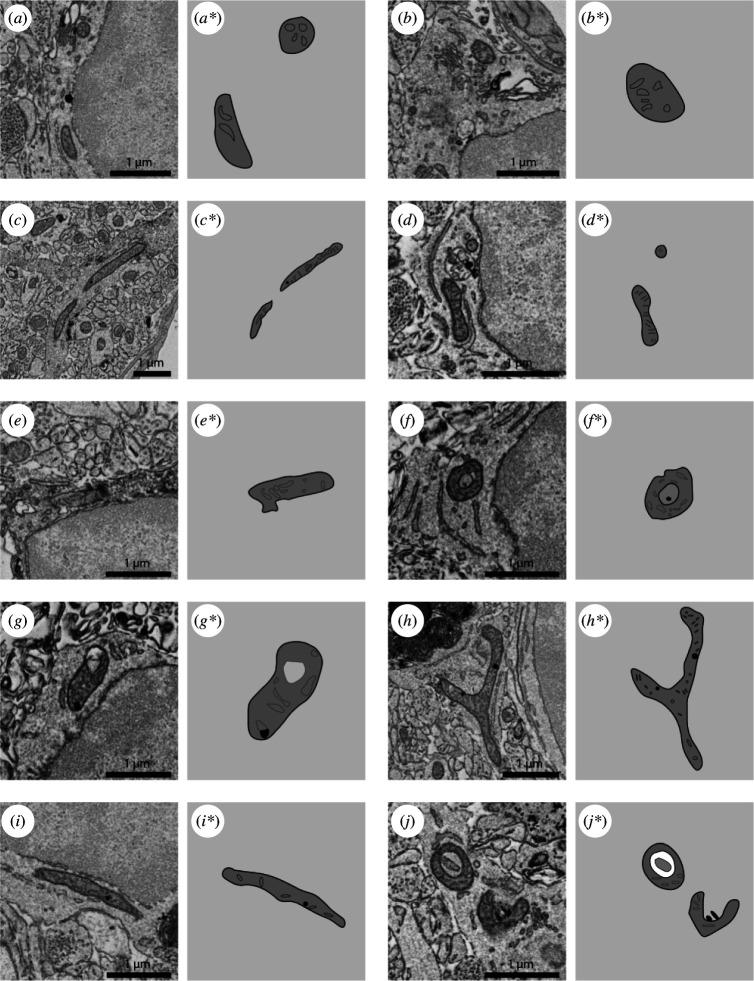
Representative 5 nm resolution scanning electron microscopy images and drawings of mitochondria belonging to hippocampal microglia of adult mice. (*a*–d*) Healthy mitochondria. (e,e*) Dystrophic mitochondria with malformations of their outer membrane. (f–j*) Altered mitochondria showing holy shape and inclusions.

**Figure 3.**
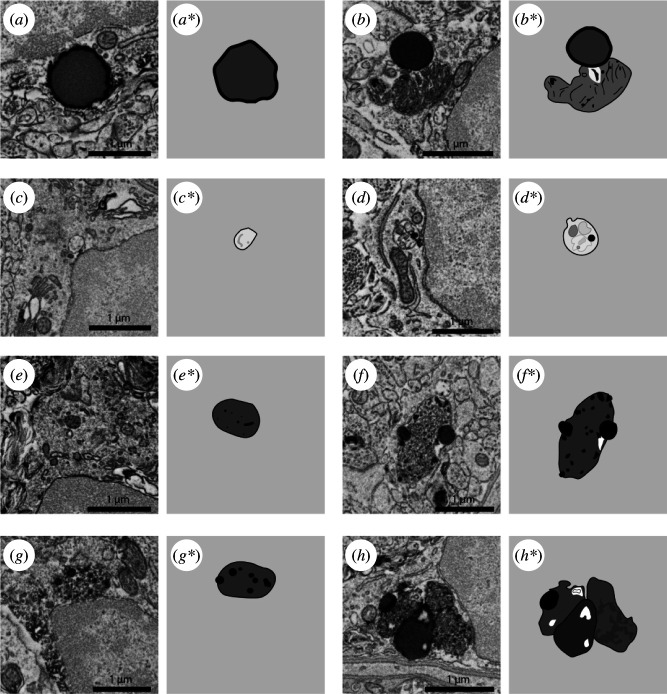
Representative 5 nm resolution scanning electron microscopy images and drawings of various organelles belonging to hippocampal microglia of adult mice. (*a,a*) Lipid droplet. (b,b*) Lipofuscin granule associated to a lipid droplet. (c–d*) Phagosomes. (e,e*) Primary lysosome. (f–g*) Secondary lysosome. (h,h*) Tertiary lysosome in association with two lipid droplets.

**Figure 4.**
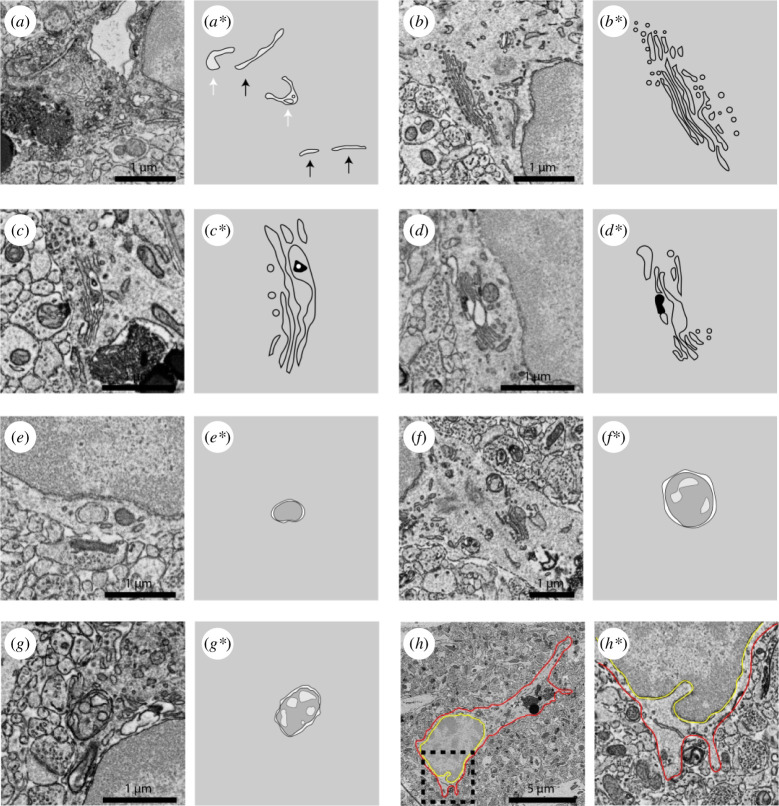
Representative 5 nm resolution scanning electron microscopy images and drawings of various organelles belonging to hippocampal microglia of adult mice. (a,a*) Endoplasmic reticulum. (b,b*) Healthy Golgi apparatus. (c–d*) Dystrophic Golgi apparatus. (e,e*) Empty autophagosome. (f–g*) Autophagosomes with content. (h,h*) Microglia showing nuclear indentation. White arrow: dystrophic endoplasmic reticulum; black arrow: healthy endoplasmic reticulum; red outline: microglial plasma membrane; yellow outline: nuclear membrane.

**Figure 5.**
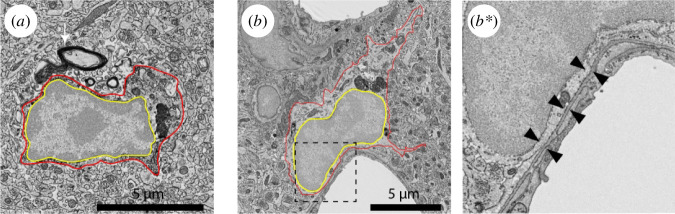
Representative 5 nm resolution scanning electron microscopy images depicting mouse microglia in the hippocampus. (*a*) Microglia contacting myelinated axons. (b,b*) Microglia contacting the basement membrane of a blood vessel. Red outline: microglial plasma membrane; yellow outline: nuclear membrane; white arrow: myelinated axon; black arrowheads: contact with basement membrane of blood vessel.

EM allows for the unique visualization of cells in their tissular environment while preserving the cell membrane and cellular organelles, often referred to as ultrastructure (figures 1–5). Using EM to study the ultrastructure of neural cells enables the quantification of relevant biological features, including the number of organelles and evaluation of the health status of such organelles [73]. Ultrastructural parameters allow the identification of specific cell types within the brain without the need for immunohistochemistry. On an electron micrograph, microglia can be identified by their round or bean shape, patchy heterochromatin pattern, long stretches of endoplasmic reticulum (ER) and high prevalence of inclusions (figure 1; [73,74]). Without staining, microglial processes are difficult to identify unless they are directly protruding from the cell body. Pairing EM with staining for the microglia and macrophage marker ionized calcium binding adaptor molecule 1 (Iba1) facilitates the identification of microglial cell bodies and processes, even those appearing discontinuous to the cell body (figure 1*a,b*). The analysis of microglial processes enables the identification and quantification of their contents and contacts within the brain parenchyma [41]. There are other resources that dive deeper in the ultrastructural identification of the other brain cells [73].

With the evaluation of resilience as a primary focus, examining the ultrastructure of organelles can be used to study the overall health status of particular cells of interest, such as microglia in the context of stress exposure [73]. This analysis helps determine the outcomes of certain treatments or environmental factors that can facilitate our understanding of the biological basis of resilience in the brain. In the following section of the review, we will focus on the organelles, their identification criteria and different ultrastructural changes providing an assessment of the physiological status of the cells and how their study can be relevant in the context of stress resilience research.

## 4. Intracellular features: organelles

EM allows the identification, classification and quantification of multiple organelles, inclusions and ultrastructural features. For instance, the ultrastructural analysis of microglia can include mitochondria, lipid droplets, phagosomes, lysosomes, lipofuscin, ER and Golgi apparatus cisternae, autophagosomes, as well as the nuclear membrane and heterochromatin pattern [75]. The next section will contain a description of the main organelles and specialized compartments that can be observed in electron micrographs of microglia. Moreover, common microglial alterations and their potential impact on cellular and brain health, as well as resilience, will be highlighted.

### 4.1. Mitochondria

Mitochondria supply energy in the form of ATP through oxidative phosphorylation, a metabolic process taking place in the mitochondrial membranes [76]. Mitochondria are double membrane organelles formed by outer and internal membranes or cristae (figure 2a-d*; [77]). Mitochondria are essential for maintaining the health of all brain cells. Increased metabolic demand, as seen following chronic stress, furthers the cumulative burden on mitochondria, which has downstream consequences, such as a compromise in metabolism and bioenergetic capacity. Brain cells and their mitochondria are known to be highly susceptible to oxidative stress [78]. Mitochondria are the main producers and the principal targets of reactive oxygen species (ROS) [76,79]. Oxidative stress can further increase ROS formation and oxidative damage [76,80]. Accumulation of ROS, mainly observed during ageing and in contexts of disease, can cause lipid peroxidation, protein oxidation and affect mitochondrial function and ultrastructure [80]. Compromised mitochondria are directly related to the viability of the cells and can serve as bioindicators for disease processes and/or disease progression [81]. Thus, the health status of mitochondria has been a focus of many therapies for brain disorders [82–86]. Recent reviews on brain disorders have pointed out that protecting mitochondria is central to a healthy brain [87]. Importantly, psychological stress has been directly linked to oxidative stress and mitochondrial dysfunction in the brain [85,88], while treatments maintaining mitochondrial health promise to provide neuroprotection against the deleterious effects of stress [88].

There are multiple ways in which EM can be used to assess mitochondrial structure and function. The primary ultrastructural alterations that indicate functional changes in mitochondria are the integrity of their outer and inner membranes, swelling of the cristae and presence of holes (figure 2e-h*; [73,89]). As the purpose of the mitochondrial double membrane structure is a maximization of their surface area, changes in the structure of mitochondria result in a modified bioenergetic capacity [90]. Mitochondria with ultrastructural alterations are considered dystrophic (figure 2e–g*, 2i–j*). Accumulation of dystrophic mitochondria can be assessed by quantifying the total number of dystrophic mitochondria per cell or by analysing the proportion of mitochondria that are dystrophic over the total number of mitochondria. Additionally, a relative proportion can be calculated by quantifying the proportion of microglia with at least one mitochondrion presenting alterations [31]. Among their distinctive features, the abundance of dystrophic mitochondria is a main identification criterion for DM. DM are also prevalent in the brain of mice subjected to repeated social defeat (RSD) stress and chronic unpredictable mild stress (CUMS) [70].

Mitochondrial fusion and fission work as opposing forces that control the shape of the mitochondrial network, and this process is important to dynamically respond to changing cellular metabolic demands [90]. Therefore, it is critical to understand that mitochondria are a dynamic network, and conventional EM captures a specific time point and a thin section of the whole mitochondrial network [91]. Thus, a single EM section only captures a fraction of the network, and mitochondrion that seem isolated are often connected [91]. Changes in the length of mitochondria, but also accumulation of longer mitochondria (<1000 nm) [31] or smaller fragmented mitochondria, provide insight into what is happening in the cell, as in the case for the activation of apoptotic pathways [92,93]. More research on the mechanisms underlying mitochondrial viability and its consequence to microglial function is expected to result in the identification of targets to improve stress resilience.

### 4.2. Lipid droplets

Cells can store lipids in lipid droplets. Lipids have various roles in processes critical for adequate cellular functioning (e.g. energy storage, signalling molecules and oxidative buffer) [94]. Under the EM, lipid droplets commonly appear round with a smooth and uniform texture, like a drop of oil on water (figure 3a–b*, 3f–h*). They can appear as either black, grey or as having a dark thick rim with a clearer colour inside. Lipid droplets can be found associated to other lipid droplets, forming lipid bodies and they can also be found associated to secondary (figure 3f–g*) and very commonly to tertiary lysosomes (figure 3h,h*). Dysregulation of lipids compromises cell function and can have negative consequences on brain health. Lipid dysregulation has been described in neurodegenerative disorders like Alzheimer’s disease (AD) or Parkinson’s disease (PD) [95]. A study described the appearance of a dysfunctional microglial state characterized by the accumulation of lipid droplets and a pro-inflammatory profile in aged mice and in the aged human brain [96]. Overloaded macrophages with lipids, known as foam cells, can affect cellular lipid homeostasis with consequences on brain health [97]. Other studies revealed that the lipid body load in astrocytes increases with environmental hypoxic stress [98,99]. Recent studies have also shown that neurons release lipid droplets that are taken up by glial cells (probably microglia and astrocytes), and protect neurons from ferroptosis, a cell death pathway driven by lipid peroxidation [100]. These results support the idea that changes in the number of lipid droplets in microglia, and other glial cells, are not only an indicator of the health status of the studied cells but the brain tissue itself. Still, the biological mechanisms underlying stress-driven lipid droplet changes continue to be understudied and require further investigation.

### 4.3. Lipofuscin

Lipofuscin results from indigestible lysosomal oxidized lipids and proteins that accumulate during ageing [101]. Lipofuscin can be identified by its fingerprint pattern and it is often associated to tertiary lysosomes (figure 3b,3b*; [102]). Lipofuscin cannot be digested, therefore its accumulation in brain cells, notably microglia, serves as an indirect way to estimate the age of an organism [101]. Because lipofuscin cannot be digested, microglia can accumulate lipofuscin in quantities which could potentially interfere with their physiological roles [103]. A partial or total reduction in the presence of lipofuscin could be interpreted as a positive feature relevant to stress resilience. A study showed an increase in lipofuscin granules in the cerebellar neurons of rats subjected to 24 and 48 h of restraint stress [104]. An increase in the number of lipofuscin granules in microglia has been reported in a mouse model of Werner syndrome, which is characterized by cellular stress and accelerated cellular ageing [105], as well as during natural ageing in mice [106]. These studies strengthen the investigation of lipofuscin abundance as a marker for ageing and stress. Better understanding of the mechanisms underlying stress-driven lipofuscin formation could provide relevant insight into the cellular and molecular pathways that modulate stress resilience.

### 4.4. Phagosomes

Phagosomes are the resulting compartments of the phagocytosis process, transporting extracellular material into the cell [107]. Phagosomes can be classified as with or without content. Both types are ultrastructurally characterized by a single membrane with a size larger than 100 nm (figure 3c–d*; [108]). Microglial phagocytic activity can be assessed using the phagolysosomal activity marker CD68 via fluorescence microscopy. Yet, CD68 does not give information on the maturation state of the organelle [109,110]. Phagosomes with content can be at an earlier stage of maturation than empty phagosomes, as the empty phagosomes are considered to have already digested the material. In some cases, the non-digested and partially digested contents can still be identified, thus providing valuable information regarding what is being internalized by microglia, such as synaptic elements [27]. Increased numbers of phagosomes of both categories can indicate higher phagocytic activity. However, the accumulation of phagosomes with content could also suggest an incapacity of the cell to digest the phagocytosed materials. Given that the origin of the phagocytic content is extracellular, identifying the cargo of phagosomes allows us to integrate the phagocytic cell in the context of its brain surroundings. This is what has allowed us to show that microglia are involved in synaptic plasticity in a sensory deprivation mouse model where the primary visual cortex receiving neuronal outputs from sensory-deprived eyes had more abundant microglia with phagosomes presenting undigested and partially digested synaptic elements still identifiable ultrastructurally [27]. Further, flow cytometry experiments showed increased numbers of microglia with a high expression of the phagolysosomal activity marker CD68 in mice exposed to RSD [111]. The authors discussed that increased phagocytosis could be used as a marker of microglia linked to stress, making the study of microglial phagocytosis using EM relevant in this context. Microglial clearance of extracellular debris can also result in an increased number of phagosomes. This is very clear in animal models of demyelination or myelin damage where there is an increase of myelin containing phagosomes as a result of the material that had to be removed [89,112]. As microglia are specialized macrophages, studying their phagosomes provides key insights into their functions in synaptic remodelling and clearance of extracellular debris, among other processes. It would be important to further study the possible involvement of microglial phagocytic processes in stress resilience.

### 4.5. Lysosomes

Lysosomes are organelles specialized in the degradation and digestion of phagocytosed materials. Lysosomes have different maturation states differentiated with EM by their ultrastructure: all maturation states are identified as single membrane structures presenting changes in their size, texture and association to other organelles. Primary lysosomes are the earliest stage of lysosome maturation. They are round in shape, are the smallest in size, have a uniform ‘salt and pepper’ texture and are not associated to other organelles (figure 3e,e*). Secondary lysosomes are bigger than primary lysosomes, they present a non-regular roundish shape, the texture is not granulated like primary lysosomes, and they can contain lipid droplets (figure 3f–g*). Tertiary lysosomes are the biggest and the most advanced in the lysosomal maturation process. They have irregular shapes and contain one or more lipid droplets of varying sizes giving them a texture of ‘oil spill over water’ (figure 3h,h*). Tertiary lysosomes are often associated with lipofuscin granules. An increase in the number of lysosomes could indicate an increased activity of the phagolysosomal system. This could be interpreted as resulting from an increase in the material that needs to be removed by microglia, from an increased phagocytic activity or an arrest of lysosomal maturation, all of these potentially could be the result of stress, ageing and pathology. Recently, it was discussed that lysosomal function goes beyond the degradation and digestion of elements. Lysosomal function has metabolic implications and even heterochromatin modification capacities [113]. The study of lysosomes in microglia can be used to study cellular processes as well as provide a direct way to obtain useful information on debris removal and elimination from the parenchyma. Studying microglial lysosomes while considering the newest literature on their function will be essential in future work assessing their involvement in stress resilience.

### 4.6. Endoplasmic reticulum and Golgi

The ER and Golgi apparatus participate in the translation, folding and modification of proteins. The ER is identified by its long membrane tubes (figure 4a,a*). The Golgi apparatus is identified as a formation of honeycomb membranes (figure 4b,b*). When ER or Golgi apparatus functions are compromised, there is an increased presence of unfolded or misfolded proteins in their cisternae. These altered proteins can then be recycled, compacted and/or sent to autophagic pathways for recycling [114]. A common ultrastructural alteration of both the ER and Golgi apparatus can be manifested as swelling of their cisternae, having a lumen bigger than 100 nm can be considered swelling, as well as the observation of precipitates within their lumen (figure 4a,a*, 4c–d*; [114–117]). Prolonged ER–Golgi stress results in apoptosis [114,118]. Accumulation of swollen ER and Golgi has been observed in microglia across pathological contexts and is a distinctive identification criteria for DM [70]. Alterations to ER and Golgi apparatus can be determined by quantifying the absolute numbers of organelles with dystrophy or by calculating the proportion of organelles that present dystrophy in relationship to the total number of ER and Golgi apparatus cisternae. A study showed that after RSD exposure, there was an increase in the expression of ER stress response proteins in the brain [119]. Another group first showed that exposing BV-2 microglia-like cells to Bullatine A, a Chinese herbal medicine, mitigated ER response to extracellular ATP exposure. Subsequently, the group revealed that microinjection of Bullatine A in the hippocampus prevented despair responses in mice subjected to RSD, suggesting that targeting the ER can potentially mitigate the cellular responses to psychological stress and increase resistance to its deleterious outcomes [120]. Further studies that provide better understanding of whether and how cellular processes involving ER and Golgi apparatus in microglia can boost stress resilience are warranted.

### 4.7. Autophagosomes

Autophagosomes are recognized as having a double membrane and a round shape with a lack of internal membrane system (figure 4e–f*). Autophagosomes are involved in the clearance and recycling of damaged molecules or organelles [121]. With EM, autophagosomes can appear empty (figure 4e,e*) or have contents of degrading materials (figure 4f,f*), including mitochondria (i.e. mitophagy). Autophagy is low at basal conditions, and may increase with cellular stress and in pathological contexts [122]. Autophagy declines with age, suggesting that the deleterious effects of cellular ageing might be owing to a dysfunctional autophagy machinery [123,124]. Proof of this comes from the fact that autophagy promoting therapies have been linked to cell survival [125]. The number and digestion stage of the autophagosomes provide valuable insight into this pathway. Molecular techniques that can detect changes in autophagy-related genes and autophagy flux [35] can be complemented by a direct quantification and characterization of autophagosomes with EM. Changes in the ratio of empty autophagosomes versus autophagosomes containing content are useful to the understanding of the digestion capabilities of cells. Other studies have shown that loss of autophagic capabilities in microglia can aggravate brain pathology [126,127] or result from neurodegeneration [128]. The lack of autophagy in contexts where autophagy is expected could potentially cause the increase of damaged organelles in microglia. Similar to phagosomes, the assessment of autophagosomes with EM allows for the quantification and characterization of these organelles and their cargo. A recent study showed increased autophagy in the hippocampus of rats exposed to CUMS [129]. The same study revealed that inhibition of autophagy was possible using bispecific (threonine/tyrosine) MAPK phosphatase 1 (MKP1) inhibition in cultured microglial cells. This study suggests that autophagy contributes to mediating resilience to the negative effects of CUMS. A previous study examining rats exposed to CUMS further indicated that chronic antidepressant treatment (fluoxetine) normalized MKP1 expression and behaviour, and that mice lacking MKP1 were resistant to stress [130]. Autophagy decline is a hallmark of cellular ageing [131], which could be linked to an increased susceptibility to the negative effects of stress owing to a decline in resilience-promoting mechanisms. This supports the importance of conducting studies focusing on microglial autophagy in the context of resilience.

### 4.8. Nuclear membrane

The nuclear envelope is a double membrane structure. Alterations in the integrity of the nuclear membrane can be presented as an accumulation of materials between the double membranes or as invaginations (figure 4h,h*). Alterations to the structure of the nuclear membrane can be a consequence of changes in their structural proteins. Irregularities associated with the nuclear envelope are a characteristic of senescent cells, which are compromised in their function owing to the accumulation of cellular stress markers yet resist cell death. These irregularities were linked to a decline in the structural protein Lamin B1 [132]. Changes to the shape of the nuclear membrane can be a consequence of changes to the cytoskeleton, as the nuclear membrane is tethered to the cytoskeleton and remodelling of the cytoskeleton results in invaginations of the nuclear membrane [133]. Mechanistic insights into nuclear membrane integrity have come from studies investigating neurons in which a collapsed cytoskeleton results in nuclear membrane invagination [134]. In homeostatic conditions, microglial cell bodies are mostly sessile and their surveillance is mostly done by the extension and retraction of their motile processes, but in some cases, microglia can migrate which would imply a cytoskeletal rearrangement [135,136]. An increased proportion of neurons presenting nuclear membrane invagination was found in vulnerable mice subjected to RSD [137], suggesting that nuclear membrane alterations can result from stress adaptations. Further studies examining the causes and consequences of nuclear invagination in microglia following stressful contexts, especially comparing between resilient and vulnerable individuals, are needed.

### 4.9. Nuclear heterochromatin pattern

An important feature used in the identification of cells at the ultrastructural level is the nuclear heterochromatin pattern [73]. Heterochromatin pattern loss has been associated with cellular stress [138] and ageing [139]. This can be observed as darkening of the nucleus, or a loss of electron density difference between the open or closed chromatin (figure 1e–e**). Changes in chromatin may indicate a reduced chromatin availability for translation which can be altered in contexts of disease and in models of stress and premature ageing [140]. The heterochromatin is tethered to the nuclear membrane [141], and loss of nuclear integrity is associated with an altered heterochromatin pattern [140]. Interestingly, heterochromatin loss has been linked to genome reorganization and aberrant translation [142]. Loss of heterochromatin pattern is a classification criterion used to identify the DM [70,143]. Determining the prevalence of microglia including DM presenting heterochromatin loss could be used to assess changes in microglial functional states during vulnerability versus resilience to stress.

## 5. Parenchymal features

An advantage of EM is the ability to see the direct contacts between different cell types. Direct intercellular contacts have been described multiple times, including between cells of the CNS. Microglia are constantly surveilling the brain parenchyma which is essential for healthy brain function [144,145]. During their surveillance, they can detect the presence of damage associated molecules and can also sense changes in brain activity [146], as those taking place in the context of stress. Microglia were found via EM to occupy satellite positions onto neuronal cell bodies where they are monitoring neuronal activity [28]. Microglia occupying satellite positions often perform synaptic stripping, by which they physically separate axon terminals and neuronal cell bodies, thereby preventing synaptic transmission [147]. Additional key components of microglial surveillance are their interactions with synaptic units, the neurovasculature and myelinated axons which are discussed below.

### 5.1. Synapses

Microglia can interact with synaptic elements via different modalities, including direct contacts. This has been observed *in vivo* [148] and through EM [27]. In two-dimensional-EM, the structures of excitatory synapses can generally be visualized in three forms: [1] pre-synaptic axon terminal only, identified by their synaptic vesicles [2]; post-synaptic dendritic spines, identified by their electron dense, post-synaptic density; and [3] synapses, identified by the presence of both pre- and post-synaptic structures and a synaptic cleft [27]. EM can be used to visualize microglia sensing synaptic activity by identifying their contacts with each of these structures (figure 1b–d*). Moreover, dendritic spines that have been directly contacted by microglia have a higher probability of disappearing *in vivo*, as shown by two-photon imaging of reporter mice in which microglia and synaptic structures are both fluorescent [27,148]. A field defining moment was the observation that microglia, by different modalities, structurally modify neuronal networks, altering neuronal transmission and contributing to plasticity during development and adulthood [27,75]. Notably, stress affects how microglia interact with synapses (extensively reviewed in [149]). In fact, microglia can eliminate complete synapses by phagocytosis [27], interrupt synaptic communication by introducing their process tips between synaptic elements (synaptic stripping) [147] and even nibble a piece of synapse in a process called trogocytosis [150]. Synaptic loss is the best pathological correlate for cognitive decline, while cognitive impairment is a common symptom of MDD. Numerous studies suggest that microglia and their particular states such as DM can contribute to pathological synaptic loss [151], notably upon stress. A reduction in synaptic density has been described in the hippocampus across multiple preclinical and clinical studies of depression [119,152–155]. Interestingly, other studies have linked this abnormal synaptic loss to engulfment by microglia [156–158]. EM was key for the understanding of microglia-driven plasticity and remains a useful tool in their study. It notably helps to link, in preclinical models, behavioural changes with structural and functional understanding of neurons, their connections and the cells able to modify their activity and plasticity [75]. Preventing or rescuing this abnormal microglia-driven synaptic elimination could be the target of future studies assessing the role of microglia in stress-related plasticity.

### 5.2. Neurovasculature

The neurovascular unit is composed of different cell types that include: endothelial cells, neurons, astrocytes, smooth muscle cells, pericytes [159] and microglia [160]. A healthy BBB is required for brain health and a disruption in the integrity of this structure can be an indication of increased permeability and entry of various mediators and immune cells into the brain. There are several features of the BBB that can be studied with EM [161,162]. BBB permeability greatly depends on endothelial tight junction proteins of endothelial cells [162,163]. Analysis of tight junctions with EM can be used to study the integrity of the BBB. Increased leakiness of the BBB has been associated to decreased levels of the tight junction protein claudin-5 only in mice developing depressive-like phenotype (i.e. susceptible, not resilient) after exposure to RSD. In line with these preclinical findings, claudin-5 expression was found to be reduced in patients with MDD. EM was used to confirm the structural consequences of these molecular changes [164]. Microglia interact directly with the BBB (figure 5b,b*), they are involved in the formation of the vasculature and modulation of the blood flow and BBB integrity [25,149,165,166]. Environmental factors can affect microglial interactions with the BBB. Microglial contacts to the BBB were shown to be affected in a sex-dependent manner in a mouse model of maternal high-fat diet [160]. DM were also seen contributing to the glia limitans of capillaries [70], but determining whether their role participates in the mechanisms of stress resilience still needs further study.

### 5.3. Myelinated axons

Oligodendrocytes produce myelin which ensheaths, insulates and provides trophic support to neuronal axons [167]. EM remains the golden standard approach for myelin integrity assessment [168]. The health status of myelin is known to be a feature of a healthy brain. Exposure to RSD caused alterations to the oligodendrocyte lineage cells which resulted in hypomyelination in mice [169]. Single nucleus transcriptomics data also revealed dysregulation in the oligodendroglial lineage cells in patients with MDD, supporting that indeed stress has an impact on this cell type. The ratio between myelin thickness and axon thickness (i.e. G ratio) is a staple of myelin analysis [170] and is used as a tool to identify changes to myelination. Myelin abnormalities or aberrations can also be quantified via bulging, redundancy or loss of compaction and outfolding [171]. The occurrence of myelin aberrations is a normal phenomenon of myelination, nevertheless, certain conditions can increase their presence, compromising brain function. Alterations of myelin structure are pathologically relevant and have consequences for plasticity and behaviour [172]. Interestingly, it was found that myelin aberrations were resolved by microglia, highlighting their important role in the myelination process [171]. Another aspect of microglia–myelin interaction (figure 5*a*) comes from the process of myelin clearance by microglia (see for review [29,173]). The study of microglial clearance of myelin is essential to understanding and promoting proper brain function [173]. Clearance of myelin by microglia is necessary for remyelination and recovery as notably seen in a demyelination cuprizone model [112]. Proper myelin clearance by microglia and remyelination could be studied as a mechanism involved in the recovery from chronic stress, resilience and the myelin alterations linked to it.

### 5.4. Three-dimensional electron microscopy

Conventional transmission EM requires the tissue to be cut into ultrathin sections that can be in the range of 65 to 75 nm in thickness. This means that we get a two-dimensional view of events that are taking place in three dimensions. Three-dimensional EM can be accomplished mainly by two different scanning electron microscopy (SEM) approaches: array tomography and focused ion beam (FIB)-SEM. Array tomography consists of acquiring images of serial sections in order to reconstruct a volume, while FIB-SEM consists of imaging the surface of the tissue and having a focused ion beam remove an ultrathin layer of the surface followed by a new image taken of the exposed surface. FIB-SEM gives the best resolution in Z, up to 3 nm, for three-dimensional EM. Array tomography’s resolution in Z depends on how thin the sections can be cut and is not destructive, unlike FIB-SEM where the tissue is destroyed. The advantage of three-dimensional EM is that it reveals detailed volumetric properties of the imaged organelles. This was demonstrated in an article that studied the mitochondrial network of cumulus cells by different imaging techniques, from fluorescence to FIB-SEM [91].

Microglia are specialized macrophages, and as discussed before, they can actively modify the neuronal circuitry using different modalities such as phagocytosis. Three-dimensional EM is required to verify whether microglia are digesting an element intracellularly, extracellularly or simply surrounding it. Three-dimensional EM is also an excellent way to complement studies on organellar communication and function that could be compromised in microglia leading to a reduction in stress resilience. Organellar function relies on constant inter-organellar communication and physical contact. These types of inter-organellar interactions are required for the creation of functional microdomains that play an essential role in communication and underlie the function of various cellular and physiological processes [174–176]. For example, a study examining a rat model of stress induced hypertension found that stress can cause dissociation of mitochondria–ER contacts, affecting calcium signalling through stress-induced inhibition of the sigma-1 receptor in microglia [177].

It is important to mention that even though two-dimensional EM was key in the discovery of microglial involvement in synaptic plasticity by phagocytosis and contributed to the study of their organelles, performing three-dimensional EM is expected to significantly refine the observations and enable a much more detailed look at microglia and their roles in resilience. For instance, this technology would allow discrimination between phagocytosis, trogocytosis, digestive exophagy and synaptic stripping thus identifying the specific mechanisms to investigate in future studies of synaptic plasticity mediating stress resilience. Applying the new advancements of EM to novel models that address the underlying mechanisms of psychological stress resilience holds a lot of potential. In the next section, we will cover nutritional ketosis as a promising therapeutic intervention to promote resilience, notably by acting on microglial ultrastructure.

## 6. Interventions that promote resilience: a focus on nutritional ketosis and microglia

Healthy lifestyle habits are known to reduce the risk of many stress-related neuropsychiatric disorders. Among them, diets that prevent or reduce the risk of disease are considered dietary interventions [178]. Ketogenic diet (KD), a low carbohydrate, moderate protein, high-fat diet, has garnered a lot of attention for its positive improvement of mood [178]. A KD forces the system to shift from carbohydrates to lipids as the main energetic source. Instead of glucose, in nutritional ketosis, energy comes from ketone bodies. β-hydroxybutyrate and acetoacetate are ketone bodies increased as a consequence of a KD or sustained fasting [179].

Exposure of rodents to sustained psychological stress is commonly used to model MDD. Changes in behaviour following exposure to this stress paradigm are often described as depressive-like (e.g. decrease sucrose preference) or anxiety-like (e.g. reduced social interaction) depending on the behavioural test. A reduction in stress-related behaviours is therefore used to evaluate the effectiveness of novel therapeutic treatments, such as nutritional ketosis. Some studies have reported that a KD is effective in reducing the negative behavioural signs of psychological stress. With an RSD mouse model that enables us to compare animal populations that are resilient versus susceptible to psychological stress [23,180,181], our recent study showed that mice following a KD had a higher proportion of individuals classified as resilient to stress compared with individuals following a control diet. Similar results were observed in another study that showed that after exposure to RSD, mice following a KD had improved social skills, reduced anhedonia and improved performance in the tail suspension test [67]. Interestingly, an early study investigated the effect of chronic and sub-chronic supplementation of ketone bodies in rats following a normal diet also showed reduced anxiety [182].

It is valuable to investigate the cellular changes that underlie improvements in behaviour. Changes in microglia have been observed in studies where KD, or supplementation with ketone bodies, resulted in increased resilience to stress. Prior studies have shown that morphological changes in microglia are representative of different functions or states of these cells. Typically, ramified, branched microglia are associated with surveilling states or homeostatic states [183]. By contrast, amoeboid shapes, with short stubby branches, have been linked to an increased phagocytic function, exacerbated release of inflammatory mediators and dystrophic states. Interestingly, increases in microglial arborization area were found to be most associated with the resilience phenotype linked to KD in adult male mice [67,181,184,185]. Another recent study showed increased microglial branch numbers and average branch length in adult male mice under a KD [186]. Interestingly this study showed that these changes were sex specific, a topic which requires further investigation.

Nutritional ketosis is also recognized for its anti-inflammatory properties [187]. Inflammation plays an important role in the adaptation to psychological stress and represents a key feature of chronic stress exposure [188,189]. This increased inflammation has therefore become a main therapeutic target for the prevention and treatment of the negative effects of psychological stress [178,187]. However, deciphering the inflammatory mechanisms that underly this process has proved to be difficult. Both the development and the resolution of inflammation are the result of a tight balance between various anti- and pro-inflammatory mediators with complex spatiotemporal relationships. Inflammation can be triggered by multiple signals beyond infection, and it plays physiological roles. An example of this comes from the activation of the NOD-, LRR- and pyrin domain-containing protein 3 (NLRP3) inflammasome owing to increased cellular stress. Cellular stress can cause an increase of misfolded proteins, which can surpass the ER unfolded protein response capacity, and in consequence triggers an inflammatory reaction via pathways such as inositol requiring enzyme 1-alpha, protein kinase R-like ER kinase and activating transcription factor 6. These responses can lead to activation of the inflammasome, and further ROS production and calcium efflux from mitochondria, which triggers a vicious loop of sustained inflammation and cellular stress [190].

KD and ketone body supplementation were shown to reduce inflammation and inflammasome in the brain, in mouse models of spinal cord injury, AD and PD pathology [190,191]. Some studies have also shown that these beneficial effects came from ketone bodies directly blocking NLRP3 mediated inflammation in the brain [185,191]. Ultrastructural evidence of cellular stress response can be assessed with enlarged ER and Golgi apparatus and the presence of luminal precipitates, features which are discussed above. A reduction of the presence of swollen ER and Golgi apparatus in hippocampal microglia was observed in adult male mice exposed to RSD and following a KD compared with a control diet [181]. These findings were hinting that KD does potentially act on microglia to reduce brain inflammation by reducing features of cellular stress.

Lastly, lipids are important molecules for brain plasticity and activity, not only as components of membranes, but also as messengers and regulators of organellar function [192]. Exposure to chronic stress causes changes to the brain lipidome in rodents [192]. Lipidomic analysis of the hippocampus further showed that brain lipids are differently regulated by KD in adult male mice susceptible to RSD [181]. These changes were different from those observed in mice classified as resilient to the same RSD paradigm [181], suggesting that stress susceptibility could be the result of distinct lipidic regulations.

## 7. Conclusion

Psychological stress is a major environmental risk factor that is present across many diseases and remains highly relevant to neuropsychiatric disorders. Resilience holds the potential to reduce the negative effects of stress and prevent its consequences, thus positively improving life quality. The biological mechanisms underlying resilience have mostly remained elusive, and the field will tremendously benefit from additional studies using novel techniques, such as three-dimensional EM, contributing to the acceleration of result translation into potentially clinical strategies.

Microglia are required for proper brain development, activity and plasticity. Understanding their role in stress resilience, as an extension of homeostasis, is expected to allow to slow down or block the negative effects of stress and prevent the emergence of stress-related neuropsychiatric disorders such as MDD. Microglia as coordinators of brain inflammation, detectors of neural activity and neural network architects, among other key roles, provide multiple options for therapeutic alternatives. Assessing the potential of emerging therapies with advanced imaging techniques is thus required.

EM provides high-resolution images of cells in their environment allowing researchers to evaluate the structure and integrity of cells, their internal components and their interactions, all key to stress resilience. Studying microglia with the help of EM will allow us to discover new pharmacological targets and better evaluate the clinical impact of new therapies and healthy life habits, such as nutritional ketosis. The increased accessibility of three-dimensional reconstructions with nanometric resolution brings together information on structure, location and function, providing valuable understanding of cellular processes involved in stress resilience.

Despite its technical complexity, EM is becoming more available thanks to new microscopes with friendlier user interphases, more automatized workflows and increased throughput. In addition, the rise of pattern recognition software and the use of neuronal networks for the identification of structures is accelerating the analysis and the automatic segmentation for the construction of three-dimensional models. There is an increased availability of published material on EM and ultrastructural characterization. This will certainly help accelerate the study of the role of microglia in stress resilience.

The study of this role of microglia in stress resilience holds a lot of potential. These cells sit at the crossroads of brain inflammation, neuroplasticity and housekeeping function. They show rapid response to tissue damage, different types of peripheral signals, changes in brain activity and environmental factors, such as diet, which makes them a promising target for novel therapies promoting stress resilience. Ultrastructural analysis of microglia emerges as a powerful tool to understand the cellular and subcellular processes involved in stress resilience, including cell–cell interactions, synaptic plasticity, phagocytic processes, direct communication between organelles and response to cellular stress.

## Data Availability

This article has no additional data.
